# Effective Multidisciplinary Search Strategies for Assistance Animals: A Librarian's Perspective

**DOI:** 10.3389/fvets.2019.00063

**Published:** 2019-03-19

**Authors:** Erik Davis Fausak

**Affiliations:** University Library, University of California, Davis, Davis, CA, United States

**Keywords:** assistant animal, service animals, therapy animals, assistive tools, disabilities, databases, subject guides, service dogs

## Abstract

Successful search strategies are based on good background knowledge and a focused clinical research question. Due to the multidisciplinary nature of research involving assistance animals means there is no one universal database to answer all research questions. The topic of assistance animals can yield better results when creating subheadings based on discipline focus. Subheadings have been divided into ethicolegal, sociocultural, psychobehavioral, and medical/veterinary. Each subheading, or discipline, has their own specific databases that will yield higher relevant content than others. Contacting local academic librarians and utilizing search guides created by those librarians can lead to successful search strategies. The goal of this article is to create a template for successful search strategies in assistance animals. Eighty-nine subject guides curated by academic librarians are reviewed to identify strong databases for each topic of ethicolegal, sociocultural, pscyhobehavioral, and medical/veterinary topics in relationship to assistance animals. A live subject guide has been created and maintained at https://www.library.ucdavis.edu/guide/assistance-animals/

## Introduction

Assistance dogs touch on all levels of academic thinking that cannot be researched with one database or search strategy. Like the Medical Subject Headings utilized by National Library of Medicine, many diverse subheadings can be incorporated into this subject to improve the sensitivity (or finding the highest amount of relevant articles in searches) (1). The study of assistance animals is highly interdisciplinary in nature because of the level of human and non-human involvement. Four major subheadings (or broader disciplines) have been identified in relationship to assistance animals: ethicolegal, sociocultural, psychobehavioral, and medical/veterinary. All of these disciplines may overlap to some degree, but this broader grouping of disciplines as subheadings can help the researcher identify ideal databases based on the penchant of their research. The goal of this paper is not to create a strict bibliography but to identify key search strategies and tools to find relevant information regarding any level of research around Assistance Animals. Whenever possible, utilizing specialized academic libraries and librarians will prove to be extremely beneficial.

## Barriers to Searching on Assistance Dogs

In models of evidence-based veterinary medicine training, it is important to develop a strong clinical question or have a developed topic. The same applies to any research. Before having a developed, or “foreground” question, it may require searching “background” information. The sources that are utilized to answer more generalized background questions will be different from someone who is well-versed in a topic and has a highly developed research question (2, 3).

Information overload can be a concern for any investigator, particularly when using search engines like Google or Google Scholar (4). A vague question will yield too many and irrelevant results, so it is important to develop a two-step strategy: A generalized inquiry to further familiarize oneself with the topic and develop a good research question which will lead to a more focused search that will yield higher relevant scholarly literature with fewer irrelevant results (2, 3).

While web search engines like Google Scholar are becoming more efficient at retrieving similar data to bibliographic databases, they still don't have the sensitivity of the more costly bibliographic databases (4–6). Freely accessible databases and search engines will be addressed for each subheading, but it is important to keep in mind that many universities (particularly land grant public universities) may allow the public to enter their library and access their resources (including the librarians) from the physical library. Additionally, many public libraries (89% of 29 scanned library homepages across the United States) can offer generalized bibliographic databases accessible from the comfort of home (7). Inter-Library Loans are also services offered by public libraries to give non-academic affiliated persons access to academic resources (8).

Encouragingly, as more journals are on an Open Access (OA) model which allows for a reader to freely access their content, more researchers have been able to find relevant literature. It is also important to keep in mind that many journals utilize a hybrid Open Access model, where some articles may be accessible while others are behind a subscription pay wall. Hybrid models have created some degree of challenge in the discovery of open access articles because they are embedded in subscription journals (9, 10).

## Generalized Search Strategies for Undeveloped Research Topics and Background Information

Asking a good clinical question is predicated on familiarity with a topic in general. A few resources can be utilized to answer background questions or get an overview of a topic. Textbooks and quality websites are certainly a good starting point. Textbook retrieval is as simple as using your library catalog and using a few keywords on the topic of interest. Searching google or any other web searching service is also a good starting point but requires judicious evaluation and selection.

An axiom of the web is that it can have quality and lackluster content in the same results. A few tools have been developed to improve the evaluation of websites. One technique is utilizing a checklist to see how the site measures up to some basic evaluative components (11, 12). Another method that may not be mutually exclusive to the checklist, is to compare websites and see if information on the website is corroborated from other sources (11–13). CRAAP represents utilizing the following criterion to use in evaluating a website (retrieved 12/20/2018 from http://www.csuchico.edu/lins/handouts/eval_websites.pdf):

- Currency – Is the topic maintained and up to date?
- Relevance – Is this information relevant to the topic you are interested in?
- Authority – Who is the author and are they qualified to write on this topic?
- Accuracy- Where is this information coming from and does it use evidence?
- Purpose – why is this paper being written? Is the author objective?

Arguments have been made that a rigid checklist is too much effort and a student can simply compare websites to identify quality differences between them and find what data is corroborated across sources (13). A caveat is that corroboration does require identification that the information came from two independent sources and not from the same one.

Performing background searches is extremely helpful in developing successful keywords. For instance, looking at an E-book on assistance animals can help identify alternative keywords: guide, hearing, service, social, support, or therapy animals. Similarly, common guide and service breeds of dogs are Labrador Retrievers, Golden Retrievers, and German Shepherds which can also help in finding search terms (14). A thesaurus (whether subject or general) can also be a useful tool for finding good keywords for searches (15).

## Understanding the Language of Searching: Boolean

**Figure 1:**
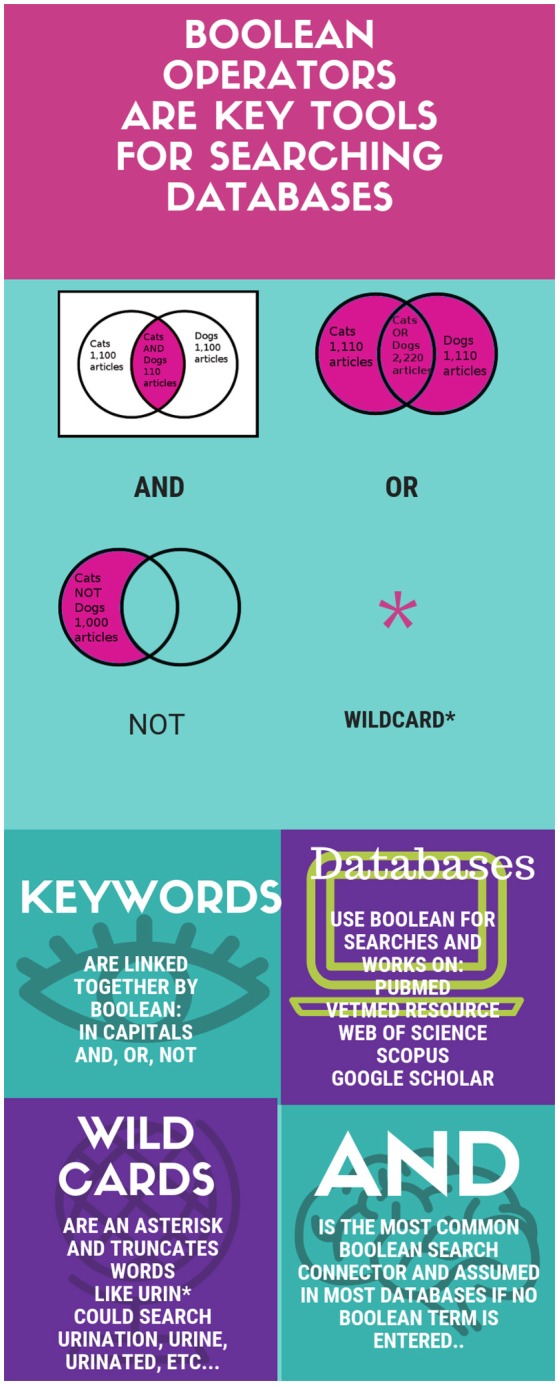
Boolean Basics. Erik Fausak (CC 2017).

Databases and search engines use similar language in combing the web or database, and this language is Boolean logic. Boolean simply takes terms, or keywords, and either looks for the appearance of them together with “AND” (which is often assumed), or it looks for any instance of any term entered with “OR” (see Figure 1). The general principle is that if a search needs to be narrowed with fewer results, use “AND, and if it needs to be widened with more results, use OR. Many search engines, like Google, use Boolean, but are more limited than many databases. Google assumes “AND,” and will use “OR” if it is written in caps between keywords. “NOT” for exclusion is represented by a—(dash) and Google can limit results by site, words in a title, url, and file type (site:, intitle: or allintitle:, inurl:, or filetype:, respectively) (16, 17). The first rule of any database or web engine being searched, is to contact a librarian or find the help icon on the database that explains what Boolean operators and other tools exist and how they can be used.

## Focused Searching (by Subheading)

Once a research question has been formed, it is time to start to focus on discovering answers to that specific question. The list below is a starting point based on many subheadings or disciplines that can be pursued. The best option is to contact a subject specialist librarian at the nearest academic library. Many University libraries are open to the public and this is a good opportunity to use its resources, including and most importantly, the librarian. Subject specialist librarians at most institutions will curate online subject guides that contain the best content tailored to the level of database access at their institution. The best approach to starting the search of a focused research question is to work with a local librarian to develop the search strategy.

## Methodology for Subject Searching

**Figure 2:**
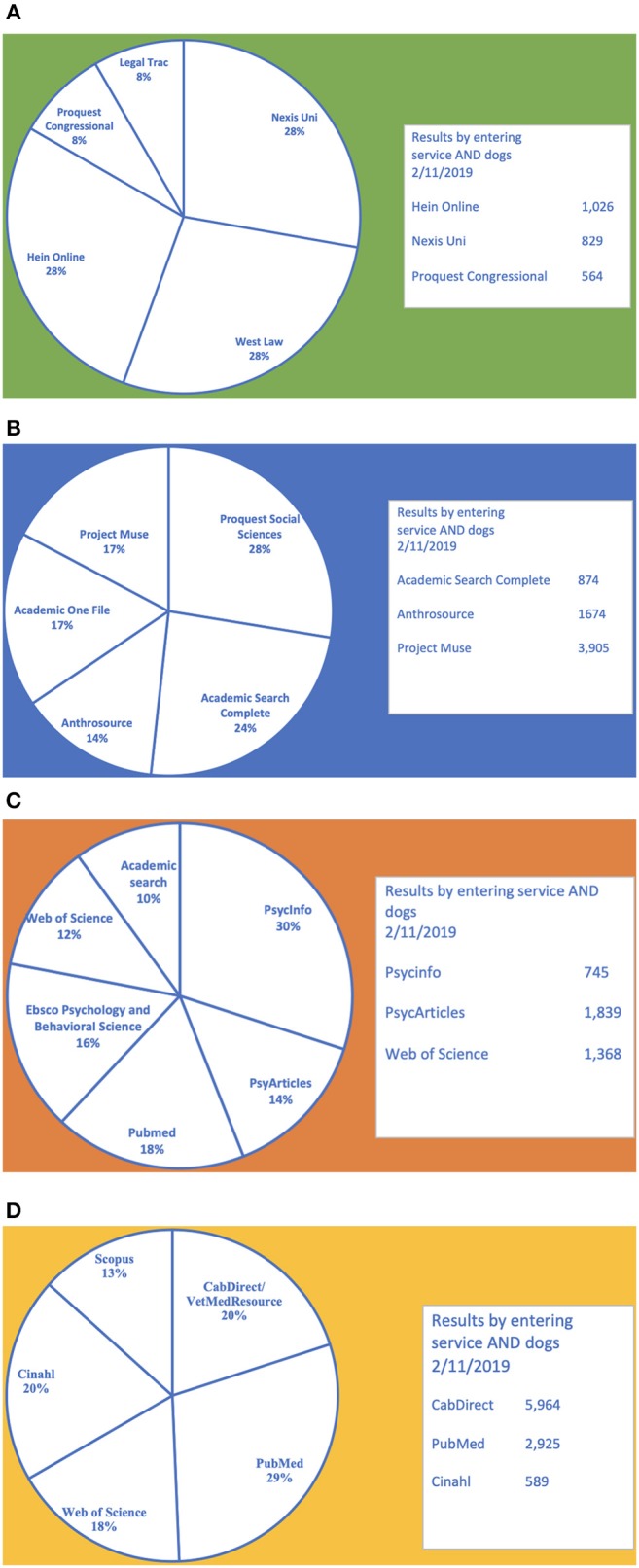
Five most frequently cited databases from 26 Sociocultural subject guides. **(B)** Five most frequently cited databases from 22 Sociocultural subject guides. **(C)** Five most frequently cited databases from 20 Psychobehavioral subject guides. **(D)** Five most frequently cited databases from 21 Medical/veterinary subject guides.

A subject guide dedicated to ethicolegal, sociocultural, psychobehavioral, and medical aspects of assistance animals will be curated and maintained at https://www.library.ucdavis.edu/guide/assistance-animals/ Twenty to thirty subject guides pertaining to the following subheadings were consulted: ethicolegal, sociocultural, psychobehavioral, and medical aspects (see Tables 1–4). Search strategies utilized in Google are recorded including authorship (if available) and last date updated when the site was being evaluated. Subject guides utilized were retrieved in order of retrieval on Google search engine results and based on relevance. The number of subject guides utilized was an arbitrary saturation point that the author felt represented a good overview of resources in the subheading area. The total number of subject guides consulted were 89 averaging 22 subject guides per subheading. Subject guides were searched and evaluated between December 21, 2018 and January 2, 2019. In lieu of a specific bibliography, the goal of this article is to identify resources for the investigator to develop their own bibliography at point and time of need. Common repetition of databases between subject guides were used to create these resource lists (see Figures 2A–D). Additionally, Three of the five databases were searched for each subheading to see what results (due to the variability in search functionality of databases, some filters were applied appropriate to the topic) occurred when searching: **service AND dogs** (see Figures 2A–D). All searches done in databases for Figures 2A–D were performed February 11, 2019.

**Table 1:** Ethicolegal guides.

**Subject guide**	**Google search term**	**Last updated**
https://guides.library.harvard.edu/animallaw	Animal law library subject guide	3/9/18
https://guides.ll.georgetown.edu/c.php?g=273353&p=1824602	Animal law library subject guide	8/9/18
https://libguides.law.uconn.edu/animal	Animal law library subject guide service	10/16/18
https://blogs.loc.gov/law/2014/07/an-introduction-to-animal-law/	Animal law library subject guide service	9/24/14
https://libguides.tru.ca/animallaw	Animal law library subject guide service	10/2/18
https://guides.sll.texas.gov/animal-law/service-animals	Animal law library subject guide service	12/27/18
http://wilawlibrary.gov/topics/disability.php#service	Animal law library subject guide service	5/8/18
https://researchguides.library.tufts.edu/c.php?g=375798&p=2543147	Animal law library subject research guide service assistance support dogs	11/2/18
https://law.duke.edu/lib/research_guide/	Law library libguide OR subject guide	various (splash page)
https://libguides.law.unm.edu/Animal	Animal law library subject guide service	9/18/18
http://libraryguides.law.pace.edu/animals	From UNM guide	7/25/18
http://libguides.law.uci.edu/c.php?g=20258&p=3080864	Animal law library subject guide service	10/18/18
https://libguides.law.uga.edu/animal_law	Animal law library subject guide service	10/15/18
https://libguides.lib.msu.edu/animalethics/generalinfo	Animal law library subject guide service	7/25/17
http://library.lclark.edu/law/animal-law	Animal law library subject guide service	10/3/18
https://libguides.stthomas.edu/c.php?g=88886	Animal law library subject guide service	8/10/17
https://libraryguides.missouri.edu/animallaw	Animal law library subject guide service	12/17/17
https://www.jenkinslaw.org/research/guides/animal-law/animal-law	Animal law library subject guide service	4/19/18
https://guides.library.ualberta.ca/c.php?g=532114&p=3640391	Animal law library subject guide service	12/19/18
https://guides.mysapl.org/servicedogs	Animal law library subject guide service	7/26/18
http://fclawlib.libguides.com/specialeducation/animals	Animal law library subject guide service	12/12/18
https://libguides.ctstatelibrary.org/dld/accessibility/ADA	Americans with disabilities act subject guide library service animals	10/9/18
http://libguides.law.berkeley.edu/c.php?g=507592	Law subject OR research OR libguide	12/3/18
https://libguides.aston.ac.uk/Law	Law subject OR research OR libguide	11/19/18
http://libguides.cdu.edu.au/cdulaw	Law subject OR research OR libguide	12/6/18
http://guides.library.cornell.edu/onlinelegalresources	Law subject OR research OR libguide	12/12/18

Included in each subheading are journals that are often referenced, but it is worth mentioning that good information comes from many sources, not just one journal (18). The cited journals are only meant to indicate good places to start browsing, but not to conduct an exhaustive search which should be performed with databases. Tools like Google Scholar's H5 score, Clarivate's Web of Science Journal Citation Reports, Scopus' Citescore, Eigenfactor, or Scimago Journal and Country Rank can all help to identify journals that are frequently cited in a particular discipline (19).

## Ethicolegal

Ethical and legal issues that surround assistant animals has become a large area of interest in recent years. Legal recognition or definition of different types of assistant animals is important to many investigators (20). Freely accessible resources to explore is Cornell University's Legal Information Institute (LII—https://www.law.cornell.edu) (Personal communication with Adam Siegal on 12/20/2018) and the Library of Congress Law Library (http://www.loc.gov/law/) that provides education and a list of important databases and e-resources. Google Scholar started discovering legal cases in 2009, which makes it a good freely usable tool for legal research. Google Scholar also has a case law filter to help search the legal literature (21). Bepress has an Animal Law Digital Commons that identifies open access (freely available) legal content from many university repositories. Recently purchased by Elsevier, there are some concerns about Bepress' continued open access role (22). Ebsco's GreenFile, and Masterfile are also useful general databases available through many public libraries to help retrieve legal literature (7). Assistance animals are covered under a specialized and rapidly growing area called Animal Law, “Under its broadest definition, animal law covers all aspects of the law—legislative, judicial, regulatory, executive—that deal with issues pertaining to non-human animals”(23). Examining 26 generalized legal research guides and Animal Law specific research guides (see Table 1). The following databases were cited the most: Thomson Reuters' Westlaw, Nexis Uni (formerly LexisNexis), and Hein Online (which has a special collection on Animal Studies: Law, Welfare, and Rights) (see Figure 2A). Additional databases for consideration are Proquest's Congressional and PAIS. Website sources that have stood out as very useful are Michigan State University's Animal Legal and Historical Center, the National Anti-Vivisection Society's Animal Law Resource Center, and governmental sites like Housing and Urban Development and Americans with Disabilities Act. Journals to follow that cover animal law include *Animal Law Review, Journal of Animal Law*, and the *Journal for Critical Animal Studies*.

## Sociocultural

A large scale multi-disciplinary approach has arisen to understand humans through their interactions with non-humans. This has given rise to the multidisciplinary efforts called anthrozoology, human-animal studies, or animal studies (24). Identifying the role of assistance animals in a larger psychological, societal, biological, humanistic, or cultural context has become increasingly important. Duke University's Evolutionary Anthropology program has developed the Canine Cognition Center that researches service dogs from an evolutionary perspective. Google Scholar poses a particular problem as recent research suggests that a great deal of social science content is still locked in subscription databases (5, 25). Examination of 22 library subject guides on anthrozoology, animal studies, and human-animal studies have yielded a great deal of resources (see Table 2). Besides Google Scholar, freely available resources include Elsevier's Bepress Digital Commons on Animal Studies and the US Department of Agriculture's Agricola database. Besides Agricola, freely available US government sites like science.gov and the Catalog of US Publications are useful resources to investigate. Many public libraries do have access to some premium access databases that have sociocultural content like Ebsco's Academic Search Complete, Gale's Academic One File and Ebsco's Greenfile (7). Premium databases at academic institutions that warrant investigation (see Figure 2B) are Wiley's AnthroSource, Proquest's Social Sciences and PsycInfo databases and JSTOR. Elsevier's Scopus and Clarivate's Web of Science, and Ebsco's Anthropology Plus also warrant consideration. Websites to explore include Animals and Society Institute, International Society for Anthrozoology (ISAZ),and H-Animal. There are many journals that explore the relationship of animals and people, which includes assistant animals. Some frequently cited journals include: *Anthrozoos, Humanimalia, Animal Studies Journal, Between the Species, Antennae*, and *Animals*.

**Table 2:** Sociocultural guides.

**Sites**	**Search terms**	**Last updated**
https://guides.lib.unc.edu/ANTH125M	Anthropology sociology human animal Research guide dogs libguide	11/15/18
https://guides.main.library.emory.edu/c.php?g=50800	Anthropology sociology human animal research guide dogs libguide	10/18/18
https://libguides.denison.edu/anthropology-sociology/articles	Anthropology sociology human animal research guide dogs libguide	12/21/18
https://uncg-lis.libguides.com/c.php?g=891820&p=6412790	Human animal bond libguide assistance animals dogs	12/3/18
https://libguides.smith.edu/ant200	Human animal bond libguide assistance animals dogs	8/6/18
https://library.ncc.edu/c.php?g=308945&p=2061646	Human animal studies libguides OR research guides	11/5/18
https://libguides.lib.msu.edu/humananimalbond/websites	Human animal studies libguides OR research guides	10/3/18
http://libguides.evergreen.edu/anthrozoology	Human animal studies libguides OR research guides	12/29/18
https://libguides.canisius.edu/c.php?g=857516&p=6143301	Anthrozoology research subject libguides	10/19/18
https://guides.libraries.wm.edu/animalstudies	Anthrozoology research subject libguides	8/16/18
https://www.carroll.edu/databases/library-databases-subject/anthrozoology	Anthrozoology research subject libguides	No date
https://libguides.rutgers.edu/c.php?g=415715&p=2835073	Anthrozoology research subject libguides	11/1/18
http://www.uwindsor.ca/anthrozoology/301/resouces	Anthrozoology research subject libguides	Not listed
https://www.canterbury.ac.nz/arts/research/nzchas/resources-and-links/	Anthrozoology research subject libguides	Not listed
https://researchguides.library.brocku.ca/c.php?g=99780&p=3125144	Human-animal studies research guide libguide	11/28/18
https://guides.nyu.edu/animalstudies	Critical animal studies library research guide OR libguide	10/30/18
https://library.barnard.edu/find-books/guides/WMST/WMSTX3513001	Critical animal studies library research guide OR libguide	Not listed
https://guides.library.ubc.ca/c.php?g=700746	Critical animal studies library research guide OR libguide	1/18/18
https://simmonslis.libguides.com/c.php?g=832520&p=5944397	Critical animal studies library research guide OR libguide	5/1/18
https://libguides.lub.lu.se/c.php?g=297124&p=1983493	Critical animal studies library research guide OR libguide	8/31/18
http://library.stanford.edu/guides/ladies-tramps-and-other-furry-friends-rhetoric-pets	Critical animal studies library research guide OR libguide	Not listed
https://researchguides.dartmouth.edu/wrt5animalstudies	Animals in literature and art libguide OR research OR study guide	6/9/17

## Psychobehavioral

A great deal of disciplinary overlap occurs between anthrozoology and psychology. Psychological aspects for consideration are the relationship between the human and assistance animal, the psychological behavior for selection of the assistance animal and their training (26, 27). Examination of 20 library subject guides on general psychology and animal behavior suggests a number of resources to find information (see Table 3). Freely available databases and search engines include Google Scholar, Pubmed, and Educational Resources Information Center (ERIC). More academic oriented resources include Proquest's PsycInfo, American Psychological Association's PsycARTICLES, Ebsco's Psychology and Behavioral Sciences Collection, Clarivate's Web of Science, and Ebsco's Academic Search Complete (see Figure 2C). Useful online resources include: Psychology Today, National Institute of Mind Health, Animal Behavior Society, Association for the Study of Animal Behavior, and the American College of Veterinary Behaviorists. Relevant journals to browse include: *Applied Animal Behavior Science, Journal of Comparative Psychology, Animal Behavior, Animal Cognition, Journal of Experimental Psychology*, and *the American Journal of Occupational Therapy*.

**Table 3:** Psychobehavioral guides.

**Sites**	**Search terms**	**Last updated**
https://www.lib.ncsu.edu/vetmed/boards/acvb	Animal behavior psychology dogs research guides or libguides	9/25/18
https://guides.library.yale.edu/c.php?g=296049&p=1973511	Animal behavior psychology dogs research guides or libguides	8/14/18
https://guides.lib.vt.edu/subject-guides/psyc	Dog psychology behavior research subject guides libguides	8/31/18
http://libguides.richmond.edu/psychology	Dog psychology behavior research subject guides libguides	4/3/18
https://sru.libguides.com/psychology	Dog psychology behavior research subject guides libguides	8/27/18
https://libguides.utk.edu/c.php?g=188662&p=1246494	Dog animal psychology research subject guide libguide	12/15/17
https://libguides.lib.fit.edu/PSY/Animal-Behavior	Dog animal psychology research subject guide libguide	12/5/18
http://mville.libguides.com/biology/Animal_Behavior	Dog animal behavior research subject guide libguide	10/1/18
https://guides.library.georgetown.edu/animalbehavior	Animal behavior research subject guide libguide	10/4/18
https://libguides.exeter.ac.uk/animalbehaviour	Animal psychology behavior research subject guide libguide	12/17/18
https://guides.library.illinois.edu/psych	Animal psychology behavior research subject guide libguide	12/7/18
http://guides.library.cornell.edu/c.php?g=31828&p=201586	Animal psychology behavior research subject guide libguide	10/25/18
http://guides.highpoint.edu/psy/home	Animal psychology behavior research subject guide libguide	12/11/18
http://libguides.ahu.edu/friendly.php?s=occupationaltherapy/animalassisted	Assistance therapy animals psychology libguide	12/21/18
https://libraryguides.missouri.edu/c.php?g=28337&p=4157952	Assistance therapy animals psychology libguide	10/5/18
https://libraryguides.lib.iup.edu/c.php?g=200983	Assistance therapy animals psychology libguide	7/24/18
https://libguides.northwestern.edu/counselingguide	Assistance therapy animals psychology libguide	12/16/18
https://amplibrary.wvwc.edu/c.php?g=521913&p=3568744	Assistance therapy animals psychology libguide	11/25/18
https://xula.libguides.com/c.php?g=203098&p=1339467	Human animal psychology behavior research subject guide libguide	9/18/18
http://libguides.mtaloy.edu/c.php?g=268088	Human animal psychology behavior research subject guide libguide	1/12/18

## Medical/Veterinary

In a broad sense, medical considerations can apply to either the assistant animal or whom the assistant animal is assisting. Occupational therapy has found assistant animals as increasingly popular and beneficial assistive tools to the disabled (28, 29). Twenty-two subject guides were examined relating to veterinary medicine and the treatment of assistant animals, general human medicine and occupational therapy in the utilization and benefit of assistant animals in human medicine (see Table 4). A number of freely available databases are utilized in both medicine and veterinary Medicine. NIH's Pubmed (Medline) is one of the best freely available resources. Google Scholar also has a high success rate in retrieving medical and veterinary related content (5). Additional freely available tools include VetSRev (an index of veterinary systematic reviews), ERIC (includes Social Service citations and occupational therapy) and Agricola (which also includes veterinary content). OTSeeker is a freely searchable database that is specifically geared for occupational therapy. Subscription databases that are most cited include CAB Direct or VetMed Resource, Proquest's PsychInfo and Nursing and Allied Health databases. Ebsco's Cinahl, Clarivate's Web of Science, and Elsevier's Scopus have also been frequently identified as important databases across the veterinary, medical, and occupational health research guides (see Figure 2D). Cab Direct has been identified as covering the most veterinary titles and vital to any veterinary search (30). Online resources that have been cited are Cornell Consultant, Best Bets for Vets, British Small Animal Veterinary Association Library, US Food and Drug Administration, Centers for Disease Control, American Occupational Therapy Association and the Veterinary Information Network. Relevant journals to browse include the American Journal of Veterinary Research, the Journal of the American Veterinary Medical Association, Journal of Veterinary Internal Medicine (OA), Veterinary Record, Journal of the American Medical Association, Lancet, New England Journal of Medicine, Nature, Science, and Occupation Therapy International (OA).

**Table 4:** Medical/veterinary subject guide.

**Sites**	**Google search terms**	**Last updated**
https://libguides.lib.msu.edu/veterinarymedicine	Veterinary medicine research subject guide libguide	11/19/18
http://guides.library.illinois.edu/mbh/vetmed	Veterinary medicine research subject guide libguide	12/5/18
https://libguides.auburn.edu/vetmed	Veterinary medicine research subject guide libguide	11/30/18
https://westernu.libguides.com/c.php?g=301185&p=2009625	Veterinary medicine research subject guide libguide	12/20/18
http://instr.iastate.libguides.com/veterinary_medicine	Veterinary medicine research subject guide libguide	8/9/18
https://libguides.cam.ac.uk/vetmed/research	Veterinary medicine research subject guide libguide	9/6/18
http://library.lmunet.edu/c.php?g=262906&p=1755977	Veterinary medicine research subject guide libguide	11/10/18
https://libguides.usask.ca/VetMed	Veterinary medicine research subject guide libguide	12/12/18
http://libraryguides.missouri.edu/veterinarymedicine	Veterinary medicine research subject guide libguide	6/21/18
https://libguides.murdoch.edu.au/vetmed/home	Veterinary medicine research subject guide libguide	10/23/18
https://www.library.ucdavis.edu/guide/health-sciences-libraries-favorites/	Health medical library research subject guide libguide	9/18/18
http://libguides.brown.edu/health	Health medical library research subject guide libguide	8/31/18
http://guides.library.ucla.edu/medicine	Health medical library research subject guide libguide	11/28/18
https://guides.library.duke.edu/subject/health-medical-sciences	Health medical library research subject guide libguide	12/18/18
http://guides.lib.usf.edu/medicine	Health medical library research subject guide libguide	11/19/18
http://libguides.library.drexel.edu/healthsciences	Health medical library research subject guide libguide	12/21/18
http://fgcu.libguides.com/occupationaltherapy/databases	Occupational therapy research subject libguide	8/29/18
https://belmont.libguides.com/ot	Occupational therapy research subject libguide	12/20/18
https://guides.library.duq.edu/ot	Occupational therapy research subject libguide	12/7/18
http://libguides.utoledo.edu/OT	Occupational therapy research subject libguide	12/10/18
https://researchguides.library.tufts.edu/c.php?g=248790&p=1657207	Occupational therapy research subject libguide	8/31/18
https://libguides.sjsu.edu/c.php?g=230321&p=1528203	Occupational therapy research subject libguide	12/19/18

## Conclusion

Effective searching and research start with identifying available resources to answer the investigator's question. The next step is whether a focused foreground question has been formed, or more background information needs to be retrieved. Background information or questions can be answered with textbooks and quality websites. Foreground or focused questions have to be answered by finding scholarly journals in reliable databases or search engines (i.e., Google Scholar). While freely available, Google Scholar is not equipped to answer all aspects of questions that the investigator may have (5). Taking advantage of the investigators closest academic library and librarian is the best first step. Public libraries are also an important resource for those without academic affiliations. Many public libraries have research databases and interlibrary loan programs with regional academic libraries. Based on the type of foreground question and which disciplines are being incorporated, there are different optimal databases.

The greatest limitation of this article is that there cannot be any prescriptive research guide for everyone. A great deal of factors influence how the research topic is approached. As multidisciplinary approaches, like anthrozoology, become more common place, it requires identifying and searching a larger breadth of unique databases. Additionally, regional and academic levels of access influence the researcher's resources and strategies. The primary goal of this article is to identify that all researchers of all levels have a number of resources at their disposal, and it starts by identifying what academic and in some cases, public libraries and librarians, are at the researcher's disposal. Additionally, very few academic libraries don't have subject guides to assist the researcher in identifying the best resources for their institution and should be utilized. Please see Supplementary Figure S1 for links of online content referred to in this article.

## Author Contributions

The author confirms being the sole contributor of this work and has approved it for publication.

### Conflict of Interest Statement

The author declares that the research was conducted in the absence of any commercial or financial relationships that could be construed as a potential conflict of interest. The reviewer MW declared a past shared affiliation, with no collaboration, with the author to the handling editor at time of review.
