# Supplementary figures and images for: Effective Multidisciplinary Search Strategies for Assistance Animals: A Librarian's Perspective
Source: Front Vet Sci. 2019 Mar 19;6:63. doi: 10.3389/fvets.2019.00063 (PMC6446476; doi:10.3389/fvets.2019.00063)

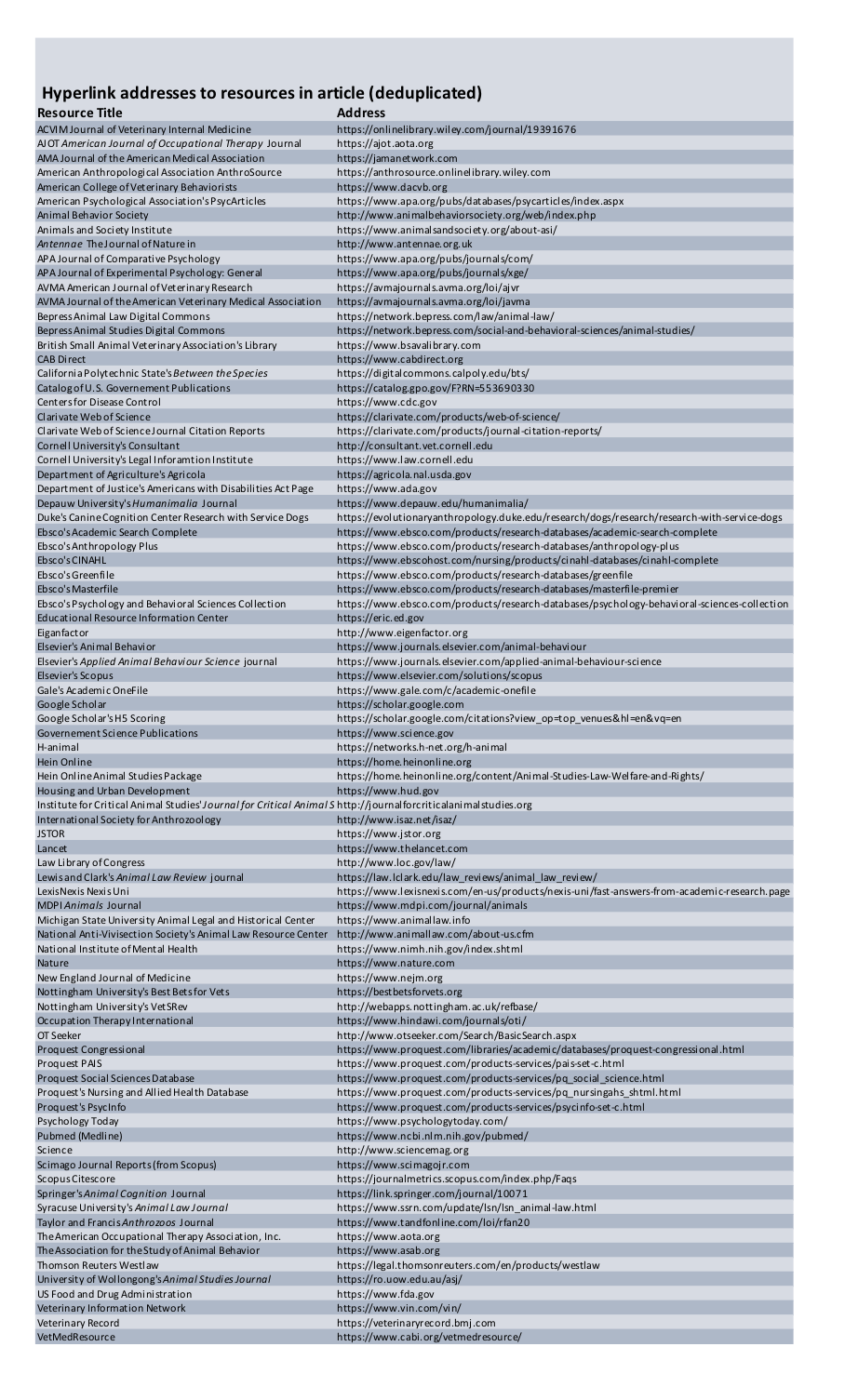

Supplement: Supplementary file 1 [file Image_1.tif]
